# Lower striatal dopamine D_2/3 _receptor availability in obese compared with non-obese subjects

**DOI:** 10.1186/2191-219X-1-37

**Published:** 2011-12-16

**Authors:** Barbara A de Weijer, Elsmarieke van de Giessen, Thérèse A van Amelsvoort, Erik Boot, Breg Braak, Ignace M Janssen, Arnold van de Laar, Eric Fliers, Mireille J Serlie, Jan Booij

**Affiliations:** 1 Department of Endocrinology and Metabolism, Academic Medical Center, University of Amsterdam, Meibergdreef 9, Amsterdam, 1105 AZ, The Netherlands; 2 Department of Nuclear Medicine, Academic Medical Center, University of Amsterdam, Meibergdreef 9, Amsterdam, 1105 AZ, The Netherlands; 3 Department of Psychiatry, Academic Medical Center, University of Amsterdam, Meibergdreef 9, Amsterdam, 1105 AZ, The Netherlands; 4 Ipse de Bruggen, Centre for People with Intellectual Disability, Zwammerdam, 2470 AA, The Netherlands; 5 Department of Gastroenterology, Academic Medical Center, University of Amsterdam, Meibergdreef 9, Amsterdam, 1105 AZ, The Netherlands; 6 Department of Surgery, Rijnstate Hospital, Wagnerlaan 55, Arnhem, 6815 AD, The Netherlands; 7 Department of Surgery, Slotervaart Hospital, Louwesweg 6, Amsterdam, 1066 EC, The Netherlands

**Keywords:** obesity, dopamine receptor availability, [^123^I]IBZM SPECT

## Abstract

**Background:**

Obesity is a result of a relative excess in energy intake over energy expenditure. These processes are controlled by genetic, environmental, psychological and biological factors. One of the factors involved in the regulation of food intake and satiety is dopaminergic signalling. A small number of studies have reported that striatal dopamine D_2_/D_3 _receptor [D2/3R] availability is lower in morbidly obese subjects.

**Methods:**

To confirm the role of D2/3R in obesity, we measured striatal D2/3R availability, using [^123^I]IBZM SPECT, in 15 obese women and 15 non-obese controls.

**Results:**

Striatal D2/3R availability was 23% (*p *= 0.028) lower in obese compared with non-obese women.

**Conclusion:**

This study is an independent replication of the finding that severely obese subjects have lower striatal D2/3R availability. Our findings invigorate the evidence for lower striatal D2/3R availability in obesity and confirm the role of the striatal dopaminergic reward system in obesity.

## Background

Over the last decades, the average body mass index [BMI] has increased worldwide. The prevalence of obesity (BMI ≥ 30 kg/m^2^) in the USA is now over 30% among adults [1]. This leads to a substantial increase in obesity-related diseases and costs. Obesity is the result of an imbalance between energy intake and energy expenditure, and these processes are normally controlled by genetic, environmental, psychological and biological factors. Excessive caloric intake of highly palatable food can be regarded as a compulsive-like feeding behaviour [2]. The mechanisms underlying a disturbed appetite regulation and overeating are poorly understood. However, a role for several neurotransmitters and hormones has been proposed (for a review, see the study of Volkow et al. [3]).

There is a large body of evidence suggesting that overeating in obesity involves the neurotransmitter, dopamine. Dopaminergic agonists induce anorexigenic effects, while treatment with dopamine D_2 _receptor [D2R] antagonists (neuroleptics) induces obesity [4]. Moreover, a high prevalence of the TaqIA A1 allele for D2R, an allele known to moderate food reward, has been found in obesity [5,6]. Finally, a role for dopamine and D2R has been established in animal models of obesity [2]. Interestingly, two imaging studies by the same group showed lower striatal dopamine D_2_/D_3 _receptor [D2/3R] availability in obese versus non-obese subjects [7,8] although in another study, a statistically significant lower availability in obese subjects was only found by a voxel-based and not by region of interest [ROI] analysis [9]. D2/3R imaging studies in obese humans are scarce and inconclusive. Therefore, we evaluated whether earlier findings of lower striatal D2/3R availability in obesity can be replicated, in order to increase our understanding on the potential role of dopamine in obesity.

## Materials and methods

### Subjects

We included 15 obese (BMI ≥ 35 kg/m^2^) women who were matched with 15 non-obese historical female controls who participated in previous studies [10,11]. Exclusion criteria for all subjects were (1) age below 18 years, (2) current or past psychiatric disease, (3) current or past exposure to dopaminergic medication, (4) lifetime history of alcohol/drug abuse, (5) concomitant or past severe medical conditions, including diabetes mellitus, and (6) pregnancy.

The 15 obese subjects are participating in an ongoing study on the early metabolic effects of Roux-en-Y gastric bypass surgery. Here, we report on the assessment of striatal D2/3R availability before surgery. Each participant gave written informed consent. The protocol was approved by the ethics committee of the Academic Medical Center of Amsterdam.

### Neuropsychological assessment

The obese subjects underwent neuropsychological assessment by the team involved in the pre-assessment for surgery and filled out the Beck Depression Inventory version II [BDI-II] for assessment of depressive symptoms.

### Single photon emission computed tomography protocol

The subjects underwent a measurement of D2/3R binding potential [BP_ND_] with single photon emission computed tomography [SPECT] and the selective radiolabeled D2/3R antagonist [^123^I]IBZM, using the sustained equilibrium/constant infusion technique [12]. The applied protocol has been described in detail previously [11]. SPECT data were acquired for approximately 60 min, starting from 120 min after the initiation of [^123^I]IBZM administration. SPECT studies were performed using a 12-detector single-slice brain-dedicated scanner (Neurofocus, Inc., Medfield, MA, USA). The obese subjects were scanned in the morning after an overnight fast; the lean subjects were scanned at various moments of the day, and they were not fasting.

### Image reconstruction and analysis

Attenuation correction of all images was performed as earlier described [13]. SPECT data were reconstructed in 3-D mode and analysed by the same investigator (BADW). For quantification, a ROI analysis was performed, with fixed ROIs for the striatum and occipital cortex, as described earlier [11]. Mean striatal and mean occipital bindings were averaged from right and left ROIs. BP_ND _was calculated as the ratio of specific to non-specific binding ((Total activity in striatum - Activity in occipital cortex)/Activity occipital cortex).

### Statistical analysis

BMI and age differences between groups were evaluated with a non-paired *t *test. Between-group comparisons in striatal D2/3R BP_ND _were performed by analysis of covariance [ANCOVA]. Since *in-vivo *D2/3R availability is influenced by natural ageing [14], age was introduced as a co-variate. Pearson's correlation coefficients were calculated with two-tailed tests of significance to investigate the relationship between striatal D2/3R BP_ND _and BMI. A probability value of 0.05 which is two-tailed was selected as significance level.

## Results and discussion

### Results

The mean BMI of the obese women was 46.8 ± 6.5 kg/m^2 ^versus 21.7 ± 2.1 kg/m^2 ^of the controls (Table 1; *p *< 0.0001). The obese women were older (37.8 ± 7.0 years) than the controls (28.0 ± 10.4 years; *p *= 0.0057). The BDI-II results showed that none of the obese women had severe depressive symptoms; only one felt in the category of mild depression (score of 14), and the others had even lower scores (scores 0 to 13).

**Table 1:** Descriptive characteristics for obese and non-obese control subjects

Descriptive characteristic	Control^a ^(*n *= 15)	Obese^a ^(*n *= 15)
BMI (kg/m^2^)	21.7 ± 2.1 (19.5 to 27.6)	46.8 ± 6.5 (38.7 to 61.3)
Age (years)	28.0 ± 10.4 (20 to 60)	37.8 ± 7.0 (26 to 49)
BDI-II score	n.a.	5.6 ± 4.2 (0 to 14)
Striatal D2/3R availability (BP_ND_)	1.12 ± 0.24 (0.75 to 1.78)	0.86 ± 0.22 (0.5 to 1.28)

^a^Data are shown as mean ± standard deviation (range); BDI-II, Beck Depression Inventory version II.

**Figure 1:**
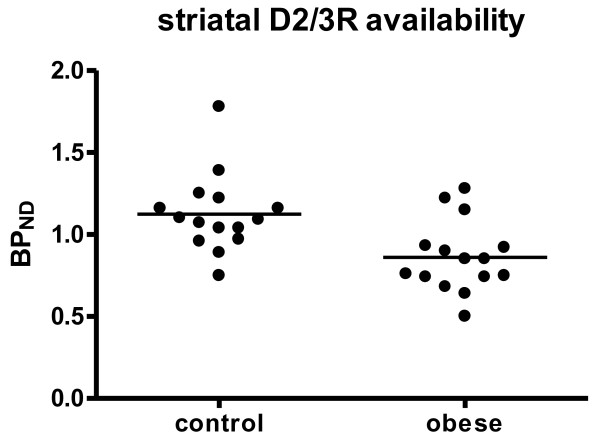
**Striatal D2/3R availability for obese and non-obese control subjects**. Horizontal line indicates mean BP _ND_.

The mean BP_ND _as a measure of striatal D2/3R availability was 23% lower in the obese group: 0.86 ± 0.22 for the obese subjects and 1.12 ± 0.24 for the controls (Figure 1). The ANCOVA revealed a significant main effect of group on D2/3R availability in the striatum (*F*(1,29) = 5.39; *p *= 0.028). There was no significant effect of age on BP_ND _(*F*(1,29) = 0.69; *p *= 0.412). The BMI did not correlate significantly with BP_ND _within the obese (*r *= - 0.392; *p *= 0.149) or the control group (*r *= - 0.141; *p *= 0.617).

## Discussion

This study replicates earlier findings that obese subjects have lower striatal D2/3R availability than non-obese subjects. The first two studies to demonstrate this difference [7,8] were in a largely overlapping sample of obese subjects with a mean BMI of 51 kg/m^2^. Haltia et al. [9] replicated this finding only with a voxel-based analysis, reporting a lower D2/3R availability in obese subjects in a cluster partly covering the striatum. The major difference with the first study was that the average BMI of the obese group was lower (33 kg/m^2^). In the present study, we included obese women with a mean BMI of 47 kg/m^2^, and we were able to replicate the finding with a ROI analysis. Thus, this suggests a decrease in striatal D2/3R availability with increasing BMI. This is strengthened by the finding of a negative correlation between the BMI and striatal D2/3R availability in the obese groups in the previous studies [7,9].

It should be mentioned that one study, performed in patients undergoing bariatric surgery, found no significant difference in striatal D2/3R availability between obese subjects and historical controls [15]. However, this study included only five women per group. Although no statistical test was described, absolute D2/3R availability shown in a graph was lower in the obese than in control subjects. Thus, this study may not have been able to detect a difference in D2/3R availability between obese and controls due to insufficient sample size.

The present study nor the previous ones can solve the question whether lower striatal D2/3R availability in obesity is a causal factor in obesity or rather the result of the obese condition. Carriers of the Taq1A allele in the gene encoding for the D2/3R show a decreased D2/3R expression [16] and have a higher susceptibility for obesity [5]. This would suggest that lower D2/3R expression levels are a pre-existing condition that plays a role in the susceptibility. However, in rats, it has been shown that downregulation of striatal D2/3R can be induced by a cafeteria diet and that this is associated with an increase in the susceptibility for reward deficits and compulsive eating behaviour [2]. The available studies on effects of weight loss after bariatric surgery on D2/3R availability are scarce and show conflicting results [15,17].

The involvement of dopamine signalling in the regulation of food intake has been clearly established [3]. Its major functions are related to motivation and reward and involvement in salience attribution to food. Food intake induces a dopamine release in the striatum thereby exerting its rewarding effect [18]. This is similar to the effects of drug abuse [19], suggesting parallels between obesity and drug addiction [3]. Part of the aetiology of both conditions could be explained by a hypodopaminergic mesolimbic system that leads to increased motivation for food and drugs, respectively [3]. In this context, it is of interest that the extent of lower striatal D2/3R availability in obese subjects is comparable to cocaine and alcohol abusers [19]. Nevertheless, lower striatal D2/3R availability is probably only one underlying mechanism in the disturbed balance between energy intake and energy expenditure present in obese subjects. Peripheral metabolic signals, e.g. leptin, ghrelin, insulin and hypothalamic neuropeptides are able to interact with the striatal dopaminergic system as well [3]. This complexity may explain the considerable overlap in striatal D2/3R availability between obese and non-obese women in the present study.

A limitation of this study is the difference in age between the obese and control subjects. To correct this, age was added as a co-variate to the statistical model. Besides, it has previously been shown that age leads to a decrease of 4.6% to 8.2% D2/3R availability per decade [14,20]. As we found a 23% lower D2/3R availability in our obese subjects, this difference is too large to be explained by age *per se*. Therefore, we believe that the age difference does not significantly affect our results and conclusions.

The two groups were not scanned under the same conditions regarding fasting state. While the obese patients were scanned after an overnight fast, the healthy controls were not scanned in the fasted state. As previously mentioned, food intake induces a striatal dopamine release [18], so this can transiently lead to increased dopamine levels. However, even if the fed state in the lean group would have led to increased dopamine levels, this would have resulted in a decrease of D2/3R availability and subsequently in an underestimation of the presently observed difference between the obese and lean groups.

Unlike previous studies on D2/3R availability in obesity with mixed gender samples, this study only included women. Although this may affect the extrapolation of the results to men, it increased the homogeneity of the subjects and demonstrated that the lower D2/3R availability is also detectable in females only.

## Conclusion

In conclusion, this study is an independent replication of the earlier finding that morbidly obese subjects have lower striatal D2/3R availability detected by ROI analysis [7]. In combination with the other available studies on this subject so far, this study invigorates the evidence for lower striatal D2/3R availability in obesity and confirms the role of the striatal dopaminergic reward system in obesity.

## Competing interests

The authors declare that they have no competing interests.

## Authors' contributions

All authors contributed substantially to the scientific process leading to this manuscript. Authors MJS, JB and EF contributed to the concept and design of the study. BADW, IMJ and AVDL acquired data on the obese subjects, and TAVA, EB and BB acquired data on the control subjects. Authors BADW and EVDG analyzed the data. EVDG and JB drafted the manuscript, which was revised by BADW, MJS and EF. TAVA, EB, BB, MJS and AVDL critically contributed to the manuscript. All authors have read and approved the final manuscript.
